# The Role of PGC1α in Alzheimer’s Disease and Therapeutic Interventions

**DOI:** 10.3390/ijms22115769

**Published:** 2021-05-28

**Authors:** Bibiana C. Mota, Magdalena Sastre

**Affiliations:** Department of Brain Sciences, Imperial College London, Hammersmith Hospital, Du Cane Road, London W12 0NN, UK; b.mota@imperial.ac.uk

**Keywords:** Alzheimer’s disease, amyloid-β, PGC1α

## Abstract

The peroxisome proliferator-activated receptor co-activator-1α (PGC1α) belongs to a family of transcriptional regulators, which act as co-activators for a number of transcription factors, including PPARs, NRFs, oestrogen receptors, etc. PGC1α has been implicated in the control of mitochondrial biogenesis, the regulation of the synthesis of ROS and inflammatory cytokines, as well as genes controlling metabolic processes. The levels of PGC1α have been shown to be altered in neurodegenerative disorders. In the brains of Alzheimer’s disease (AD) patients and animal models of amyloidosis, PGC1α expression was reduced compared with healthy individuals. Recently, it was shown that overexpression of PGC1α resulted in reduced amyloid-β (Aβ) generation, particularly by regulating the expression of BACE1, the rate-limiting enzyme involved in the production of Aβ. These results provide evidence pointing toward PGC1α activation as a new therapeutic avenue for AD, which has been supported by the promising observations of treatments with drugs that enhance the expression of PGC1α and gene therapy studies in animal models of AD. This review summarizes the different ways and mechanisms whereby PGC1α can be neuroprotective in AD and the pre-clinical treatments that have been explored so far.

## 1. Introduction

Peroxisome proliferator-activated receptor (PPAR) gamma coactivator 1 (PGC1) is a group of transcriptional regulators for a variety of transcription factors and nuclear receptors, which consists of three subtypes, PGC1α, PGC1β, and the PGC-related coactivator (PRC) [1]. Since the early 2000s, PGC1α has attracted interest because of its important role in metabolic processes (including gluconeogenesis, glucose transport, and fatty acid oxidation), mitochondrial biogenesis, peroxisomal remodelling, and detoxification of reactive oxygen species (ROS) [2]. These effects are mediated through the regulation of a number of transcription factors, including nuclear respiratory factors (NFRs) NRF-1 and NRF-2 (interacting with Tfam, which drives transcription and replication of mtDNA), PPARs (PPARα, PPARδ/β, and PPARγ), thyroid hormone, glucocorticoid, oestrogen, and ERRs (oestrogen-related receptors) α and γ [3] (ERRα, ERRβ, and ERRγ), initiator element binding factor (YY1), myocyte-specific enhancer factors (MEF-2A, MEF-2C, MEF-2D), forkhead box O1 (FOXO1), and others [4].

PGC1α was first identified in the adipose tissue, where it mediates the shift of white adipose tissue into a brown-fat-like phenotype. Therefore, PGC1α is highly expressed in tissues with elevated energy requirements, including adipose tissue, the liver, skeletal muscle, cardiac myocytes, the kidneys, and the brain [5,6]. Alterations in the levels of PGC1α have been linked with pathologies such as metabolic syndrome and its principal complications including obesity, type 2 diabetes mellitus, cardiovascular disease, and hepatic steatosis [7].

Many signalling pathways have been proposed to regulate PGC-1α expression and activity, including calcium signalling and second messengers, cyclin-dependent kinases, and post-translational modifications, such as phosphorylation, methylation, and deacetylation, and others [3]. In particular, PGC1α levels can be modulated by fasting, physical exercise, inflammation, and drugs that affect the pathways mentioned above. Exercise induces upregulation of PGC1α in skeletal muscle, where it stimulates the expression of FNDC5 and induces the transcription of BDNF [8], by increasing the phosphorylation of PGC1α by AMP-K. Fasting induces the expression of sirtuin-1, which has been shown to mediate the deacetylation of PGC1α [9,10,11]. In this regard, PGC1α has been reported to be involved in the exercise and fasting regulation of autophagy and the unfolded protein response (UPR) [12,13].

Previous studies suggested that PGC1α in the brain is enriched in inhibitory interneurons and required for the expression of the calcium buffer parvalbumin (PV) in the cortex [14]. Conditional knockout of PGC1α in the central nervous system (CNS) has revealed limited alterations in metabolic processes and its involvement in the regulation of a different category of genes linked with brain activity, including synaptotagmin 2, complexin 1, and interneuron genes [15,16,17,18]. Therefore, it appears that the functions of PGC1α in the brain are different from peripheral tissues. PGC1α overexpression was also found to protect neural cells in culture from oxidative-stressor-mediated death [19], and increase the formation and maintenance of dendritic spines in hippocampal neurons, while the opposite effect was observed in PGC1α knockout neurons [20]. In addition, adult conditional PGC1α knockout mice resulted in the loss of dopaminergic neurons, which was accompanied by a reduction in dopamine in the striatum [21]. PGC1α is also expressed in glial cells, such as astrocytes, regulating neuroinflammation and oxidative stress [22].

In neurodegeneration and brain injury, PGC1α can promote neuronal survival by affecting the activity of NFR-2 [23]. PGC1α deficiency affects mitochondrial structure and promotes mitochondrial ROS levels, leading to cellular senescence and ageing-related disorders [24]. PGC1α expression has been reported to be altered in neurodegenerative disorders such as amyotrophic lateral sclerosis, Huntington’s disease, Parkinson’s disease, and multiple sclerosis [25], leading to mitochondrial defects and increased ROS levels [26,27,28].

In this review, we focus on the role of PGC1α in Alzheimer’s disease (AD), particularly the promising treatments based on its activation.

## 2. PGC1α in Alzheimer’s Disease

AD is a neurodegenerative disorder characterized by memory and neuronal loss and the deposition of amyloid-β (Aβ) plaques and neurofibrillary tangles of hyperphosphorylated tau in the brains of the patients. Aβ is generated by the sequential cleavage of the amyloid-precursor protein (APP) by two enzymes, β-APP cleaving enzyme (BACE1) and the γ-secretase complex, whose main catalytic domain relays on presenilins (PS) [29]. Animal models used for research include generally transgenic mice overexpressing the human APP form with familiar mutations or human tau mutations [30].

Several groups have reported changes in PGC1α expression in the brain of AD patients and animal models of amyloidosis. PGC1α protein levels were reduced in brains of the Tg2576 (overexpressing APP with the familiar Swedish mutation) and APP/PS1 (which also include presenilin mutations) mouse models [31,32], as well as in nuclear extracts from human AD patients [33]. In agreement, Qin et al. reported that the mRNA levels of PGC1α decreased in the AD brain and correlated with the levels of AD dementia and Aβ pathology [34].

The role of PGC1α in the pathology of AD has been associated with reductions in Aβ levels [31,33]. Conversely, crossing Tg2576 mice with mice deficient in PGC1α or silencing PGC1α using siRNA transfection in neuronal cells led to increased Aβ [33,35]. In line with this, studies of double transgenic PGC1α and Tg19959 (containing the Indiana and Swedish mutations) mice revealed reductions in the expression of Aβ40 by ELISA;, however Congo red staining for aggregated Aβ was increased [36].

**Figure 1:**
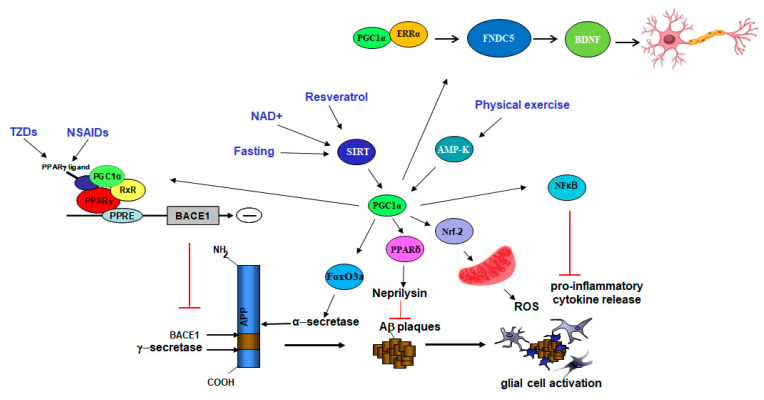
Model showing the mechanistic effects of PGC1α as therapy in AD. Activation of PGC1α via interventions (in blue) such as exercise, fasting, or treatments (genetic and pharmacological) can lead to neuroprotection in AD by targeting different transcriptional pathways. Binding to PPARγ results in changes in the processing of the amyloid-precursor protein (APP) by reducing BACE1 transcription and Aβ generation. PGC1α can affect Aβ degradation by increasing neprilysin activity. In addition, the expression of neurotrophic molecules, such as sAPPα (by increasing α-secretase expression) and BDNF, are enhanced by PGC1α. Lastly, the levels of pro-inflammatory cytokines and reactive oxygen species (ROS) are also modulated by PGC1α. Adapted from Katsouri et al., 2016 (reference [38]).

The most likely mechanism whereby PGC1α decreases the generation of Aβ seems to be by reducing the expression of the rate-limiting enzyme for Aβ production BACE1 [33,37,38] (Figure 1). In vitro, PGC1α overexpression was able to reduce BACE1 transcription and BACE1 promoter activity and the opposite effects were observed in cells transfected with PGC1α siRNA. In addition, these effects were mediated by peroxisome proliferator-activated receptor gamma (PPARγ) [33,37], since they were not detected in PPARγ-deficient cells. We previously reported that PPARγ is a repressor of BACE1 [39] and we and others found that BACE1 promoter contains PPRE domains [37,39]. However, other studies suggest that PGC1α activation may affect BACE1 proteasomal degradation through CF(Fbx2)-E3 ligase gene expression [31].

Another mechanism by which PGC1α can be beneficial in AD is by affecting the non-amyloidogenic pathway. Overexpression of PGC1α via viral vectors using primary cultures of Tg2576 mice resulted in an increase in α-secretase activity [34] via suppression of FoxO3a. Increases in the levels of non-amyloidogenic soluble APPα were also detected in N2a mouse neuroblastoma cells transfected with PGC1α cDNA, although no changes in ADAM-10 expression were observed [33]. Interestingly, ADAM-10 transcription was found to be regulated by PPARα, although experiments in mouse hippocampal neurons showed that activation of PPARα induced the recruitment of PPARα to the ADAM-10 promoter without the presence of PGC1α [40].

Further studies also considered PGC1α in Aβ degradation. Transfection of PGC1α led to an increase in neprilysin activity, but not in its expression. In addition, incubation with the PGC1α activator resveratrol increased neprilysin activity [33]. Interestingly, PPARδ agonists were found to elevate neprilysin transcription in animal models of AD [41], and neprilysin promoter contains two PPRE domains [42]. PGC-1α has also been found to mediate neuroprotective effects by protecting against Aβ neurotoxicity in N2a cells [43] and in astrocytes [44], as well as by reducing neuroinflammatory cytokines [45] and the release of ROS [19].

All these results point to the potential neuroprotective effects of the activation of PGC1α in AD. In the following sections, the pre-clinical studies performed using treatments that directly or indirectly activate PGC1α as well as gene therapy studies are analysed.

## 3. Therapeutic Effects of Activation of PGC1α in Alzheimer’s Disease

Over the years, studies have demonstrated that PGC1α can be modulated by both pharmacological and non-pharmacological approaches. For instance, PGC1α expression can be induced by exercise, fasting, and cold exposure [46,47,48]. Pharmacological activation of PGC1α can be achieved using compounds and drugs, including resveratrol and PPARγ agonists [49,50]. In addition, gene therapy approaches showed promising results in AD models [38] but small benefits in clinical trials (Table 1 and Table 2).

### 3.1. Non-Pharmacological Approaches

Gene therapy studies from our laboratory demonstrated that PGC1α gene delivery using lentiviral vectors in the APP23 model of amyloidosis (overexpressing APP with the Swedish mutation) at pre-symptomatic stages of AD resulted in decreased Aβ plaques, neuronal loss, and improved memory [38]. PGC1α overexpression in the cortex and hippocampus of APP23 mice led to decreased expression of BACE1, without changes in the mechanisms of amyloid degradation or in mitochondrial markers. In addition, PGC1α-injected mice showed reduced inflammatory markers and neuronal loss in pyramidal neurons of the CA3 area of the hippocampus and improved spatial and recognition memory compared with control APP23 mice, associated with an increased expression of neurotrophic factors. The effects on factors such as BDNF can be mediated through the PGC1α/FNDC5/BDNF pathway [8].

Recently, PGC1α was shown to display beneficial effects by regulating the expression of vitamin D receptors. Overexpression of PGC1α in APP/PS1 mice by hippocampal injection of AAV-PGC-1α resulted in an increase in the expression of VDR and a decrease in the levels of Aβ plaques [32].

Thus, there is now substantial evidence indicating that modulation of PGC1α levels in the brain may be an effective approach, although the type of viral vectors used, as well as the brain area targeted, are critical in order to obtain the expected beneficial effects. Too much overexpression of PGC1α can lead to damaging effects in particular cell types, such as dopaminergic neurons, which are more prone to degeneration [56].

### 3.2. Pharmacological Approaches 

#### 3.2.1. Resveratrol

Resveratrol is a polyphenol produced in several plants, especially grape skin and seeds. Accumulating evidence has highlighted the neuroprotective effects of resveratrol in neurodegenerative diseases, such as AD [57,58]. Special attention has been focused on resveratrol due to its multiple biological properties, including its antioxidant, anti-inflammatory, and neuroprotective effects [59,60].

Among the mechanisms underlying the neuroprotective effects exerted by resveratrol on AD, studies have suggested the activation of AMP-activated protein kinase (AMPK) and the indirect activation of silent information regulator 1 (SIRT1), as a critical pathway on AD [51,61]. SIRT1 is a nicotinamide adenosine dinucleotide (NAD)-dependent deacetylase that regulates the activity of several proteins by removing acetyl groups from them, including PGC1α [49]. In vitro, resveratrol was reported to have a potent anti-amyloidogenic activity, reducing the levels of Aβ in N2a and HEK293Tcells expressing human Swedish mutation APP_695_ by promoting Aβ clearance but not affecting APP processing [41]. Furthermore, as described in the previous section, we showed that incubation of N2asw cells with resveratrol increased neprilysin activity, yet no changes were observed in the expression or in the mRNA levels of the enzyme, indicating that PGC-1α effects on neprilysin activity may be linked to transcriptional-independent mechanisms [33]. In vivo, oral treatment with resveratrol significantly reduced Aβ levels and deposition in the cortex of APP/PS1 mice through activation of AMPK, confirming not only the anti-amyloidogenic potential of resveratrol, but also its ability to cross the blood-brain-barrier [51]. However, a 52 week randomised phase 2 clinical trial of resveratrol in individuals with mild to moderate AD detected a low concentration of resveratrol in cerebrospinal fluid (CSF), although a high daily dose of oral resveratrol was administrated, suggesting a poor bioavailability of oral treatment with resveratrol in humans [62]. However, it was still effective in stabilising the decline in CSF and plasma Aβ40 levels and attenuating the decline in a functional measurement test [62]. Although several studies have indicated the protective involvement of resveratrol in the pathophysiology of AD, more studies are required to determine the bioavailability of SIRT1 activators, such as resveratrol.

**Table 2:** Clinical trials with treatments targeting PGC1α in Alzheimer’s disease.

Treatment Affecting PGC1α (Dose)	Subject	Benefits on AD	Ref.
Resveratrol (500 mg orally once daily)	**Clinical trials** Mild to moderate AD (n = 119)	mall functional benefits	[62]
Rosiglitazone (4 mg orally once daily)	MCI or mild AD (n = 30)	Small functional benefits	[63]
Rosiglitazone (2, 4 or 8 mg daily)	Mild to moderate AD (n = 511)	Small cognitive benefits in the ApoEe4-treated group	[64]
Pioglitazone (15 mg daily)	Mild to moderate AD (n = 29)	No benefits	[65]
Ibuprofen (400 mg twice daily)	Mild to moderate AD (n = 132)	No benefits	[66]
Indomethacin (100 mg daily)	Mild to moderate AD (n = 51)	No benefits	[67]
Naproxen (220 mg once daily)	Mild to moderate AD (n = 40)	Small functional and cognitive benefits	[68]

#### 3.2.2. Nicotinamide Riboside

Nicotinamide riboside, the precursor of NAD^+^, has been reported to increase PGC1α levels through NAD-dependent deacetylase SIRT1. NAD levels have been associated with reductions in Aβ toxicity in AD models [69,70]. Pharmacological stimulation of PGC1α synthesis with 250 mg/kg/day of nicotinamide riboside, the precursor of NAD^+^, for 3 months resulted in reduced Aβ levels and attenuated cognitive deterioration in Tg2576 mice. These changes were associated with reduced BACE1 expression [35].

#### 3.2.3. Sildenafil

Sildenafil (Viagra), a drug used to treat erectile dysfunction and pulmonary arterial hypertension (particularly at low doses), likely activates PGC1α by affecting sirtuin-1 activation and PGC1α deacetylation. In transgenic mice, sildenafil appeared to reduce neuroinflammatory markers, increase neurogenesis, and improve behaviour, without evident changes in amyloid deposition [71].

#### 3.2.4. PPARγ Agonists

As indicated above, the main transcription factor regulated by PGC1α is PPARγ, and the effects of PGC1α on BACE1 expression are controlled by PPARγ [33]. PPARγ activators comprise different groups of drugs, including thiazolidinediones (TZDs) and certain nonsteroidal anti-inflammatory drugs (NSAIDs), such as ibuprofen, naproxen, and indomethacine [72,73].

TZD drugs are synthetic agonists of PPAR-γ and are the most potent activators than any endogenous ligand of PPARγ, including rosiglitazone, troglitazone, and pioglitazone. The anti-inflammatory actions of pioglitazone occur, in part, through suppression of NF-kβ and the sequestering of co-activators necessary for inflammatory gene activation. Short-term treatment with pioglitazone suppressed neuroinflammation and decreased mRNA and protein level of BACE1 in APPV717I-transgenic mice (overexpressing APP with the V171I mutation) [50]. Furthermore, long-term treatments with pioglitazone in animal models of AD resulted in reduced amyloid deposition and neuroinflammation and ameliorated learning and memory in the 3xtg-AD model [52,53]. Similarly, chronic treatment of Tg2576 and J20 mice with rosiglitazone, a high-affinity PPARγ agonist, rescued memory impairment concomitant with a reduction in cortical Aβ levels and Aβ plaque deposition [54,55]. Although TZDs have demonstrated beneficial effects in animals models of AD, clinical trials have only reported mild improvements in memory, due to the low permeability of these compounds (see [72] and Table 2).

NSAIDs were first postulated to protect from AD due to the extensive benefits in counteracting neuroinflammation through cyclooxygenase (COX) mediated inhibition [74]. Lately, additional attention was paid to certain NSAIDs, such as ibuprofen or indomethacin, due to their protective effects on AD pathology by lowering Aβ peptide levels, independent of COX activity [75,76]. NSAIDs treatments have been widely tested in animal models of AD and its effects on Aβ levels and deposition are likely dependent on the length of the treatment [77]. For example, acute-fed-treatment with ibuprofen (62.5 mg/kg/day) resulted in decreased microglial activation and slight reduction in BACE1 levels and Aβ deposition in the brain of APP transgenic mice [50]. Conversely, chronic treatment with ibuprofen or indomethacin led to a more robust reduction in amyloid deposition and activated microglia, and a decrease in inflammatory mediators in mouse models of AD [29,72]. Long-term treatment with high doses of ibuprofen (56 mg/kg and 62.5 mg/kg), sufficient to activate PPARγ, delayed neuroinflammation and Aβ deposition in the Tg2576 mouse model for AD and in vitro [52,78]. However, both drugs, ibuprofen and indomethacin, failed to demonstrate efficiency in slowing the progression of AD in patients with mild to moderate AD, in 12 month randomised clinical trials [66,67]. Naproxen, another well-established NSAID, is a non-selective COX inhibitor known to reduce inflammation through inhibiting prostaglandin synthesis and activating PPAR-γ [79]. In vivo, early treatment reduced inflammatory response, without affecting APP processing and Aβ metabolism in APP transgenic mice model of AD [80]. Evidence suggests that NSAIDs use is more effective at preventing AD before disease onset. For instance, naproxen underwent a clinical trial to assess its ability to slow cognitive decline in patients with mild to moderate AD; however, it was discontinued due to lack of efficiency [68].

## 4. Conclusions

In conclusion, PGC1α activation, either via drugs that increase its levels (such as resveratrol or nicotinamide riboside) or the activation of transcription factors regulated by PGC1α (such as PPARγ agonists), results in reductions in Alzheimer pathology and improvements in behaviour. However, although gene therapy approaches appear promising, this approach should be taken with caution, because the procedures for gene delivery are highly invasive and high overexpression of PGC1α may result in deleterious effects.

Future studies on PGC1α-based therapies should investigate the effect of other pathological hallmarks present in AD brains such as tau pathology. In addition, it would be worth exploring the effect of PGC1α activation in ageing and in animal models at late stages of the disease. The potential therapeutic value of this molecule at these stages is based on the effect in the expression of growth factors, such as BDNF, which can affect neurogenesis and protect against neuronal loss, as well as the potential anti-inflammatory effects of PGC1α.

## Figures and Tables

**Table 1:** Treatments targeting PGC1α in Alzheimer’s Disease in vitro and in vivo.

Treatment Affecting PGC1α (Dose)	Method	Outcome on AD	Ref.
Resveratrol (20–40 µM) Resveratrol (100 µM)	**In vitro** Hek293 and N2a cell lines expressing APP695 N2a cell lines expressing APP695	↓ Aβ, promoting clearance ↔ APP processing ↑ Neprilysin activity	[33,41]
Gene therapy (lentivirus carrying hPGC1α)	**In vivo** APP23 mice (8 months old)	Improved memory Rescued neuronal loss ↓ Aβ and BACE1 expression ↑ BDNF and NGF levels	[38]
Gene therapy (AAV carrying PGC1α)	2xTg-AD mice (6 months old)	↓ Aβ and ROS	[32]
Resveratrol (diet with 0.35% resveratrol)	APP/PS1 mice (4 months old)	↓ Aβ deposition	[51]
Nicotinamide riboside (250 mg)	Tg2576 mice (8 months old)	↑ PGC1α expression Improved memory ↓ BACE1 expression and Aβ	[35]
Pioglitazone (40 mg/kg/day) and Ibuprofen (62.5 mg/kg/day)	APPV717I transgenic mice (10 months old)	↓ BACE1 expression and Aβ ↓ Glial activation	[50]
Pioglitazone (20 mg/kg/day) and Ibuprofen (62.5 mg/kg/day)	Tg2576 mice (11 months old)	↓ SDS-soluble Aβ42 and Aβ40	[52]
Pioglitazone (18 mg/kg)	3xTg-AD mice (10 months old)	Improved memory Enhanced long-term plasticity ↓ Aβ and tau deposition	[53]
Rosiglitazone (5 mg/g/day)	J20 mice (9 months old)	Improved memory ↓ Aβ deposition ↓ Neuroinflammation	[54]
Rosiglitazone (diet of 30 mg/kg)	Tg2576 mice (4 months old)	Enhanced learning and memory ↓ Aβ levels ↑ IDE mRNA and activity	[55]

↑ = increased, ↓ = decreased, and ↔ = no changes.

## Data Availability

Not applicable.
